# Health promotion in the Danish maritime setting: challenges and possibilities for changing lifestyle behavior and health among seafarers

**DOI:** 10.1186/1471-2458-13-1165

**Published:** 2013-12-11

**Authors:** Lulu Hjarnoe, Anja Leppin

**Affiliations:** 1 Centre of Maritime Health and Society, Institute of Public Health, University of Southern Denmark, Niels Bohrs Vej 9, 6700 Esbjerg, Denmark; 2 Unit for Health Promotion Research, Institute of Public Health, University of Southern Denmark, Niels Bohrs Vej 9, 6700 Esbjerg Denmark

**Keywords:** Seafarers, Maritime, Lifestyle, Health promotion intervention, Workplace health promotion, Health education, Follow-up, Structural intervention

## Abstract

**Background:**

Seafaring is a risky occupation when compared to land-based industries as incidence rates of mortality and morbidity are higher. This trend is partly due to a higher number of accidents but also higher incidence of lifestyle-related diseases like cardiovascular disease and lung cancer. In Denmark, the proportion of smokers as well as of overweight and obese persons is higher among seafarers compared to the general population. This high burden of risk indicates that this occupational group might be a growing challenge at sea in regard to safety and health issues and there is a need to further our understanding of the health promotion approaches that work.

**Methods:**

A single-group pre-post design was conducted in 2008–2009 in order to identify changes in lifestyle related behaviors and health risk factors among seafarers (N: 606) in two Danish shipping companies after implementing two structural health promotion interventions (healthy cooking courses for ship cooks and improvement of fitness facilities) as well as health education interventions (smoking cessation courses, individual exercise guidance and extra health check-ups) at the maritime workplace. Baseline and follow-up data were collected with a self-administrated standardized questionnaire and individual health profiling assessing parameters such as physical health and physical fitness. In addition, qualitative interviews with participants and non-participants were conducted in order to gain in-depth information on experiences with the intervention processes.

**Results:**

Significant changes were identified for levels of fitness, daily sugar intake and metabolic syndrome. However, these results were not associated with participating in the health educational interventions. One possible explanation for the improved fitness rate could be the upgrading of fitness equipment onboard the ships provided by the management level. The decrease in daily sugar intake and prevalence of seafarers with metabolic syndrome might be associated with the cooking course intervention which aimed at providing healthier daily meals on board.

**Conclusion:**

The findings suggest that a multicomponent health promotion intervention program has the potential to achieve change in seafarers’ health behavior and health parameters. In the future, studies with more rigorous designs, separately testing the contribution of different types of interventions are needed.

## Background

Lifestyle behaviors, such as smoking, consuming foods high in fat and sugar, being overweight and physical inactivity constitute the key modifiable risk factors for many chronic health problems, among them in particular cardio-vascular diseases (CVDs), the number one cause of death globally [1]. Different population subgroups are, however, differentially afflicted and beyond factors such as age, gender, socio-economic background or education, type of occupation seems to be associated with different levels of risk. One group which has been shown to have a particularly high cardiovascular risk factor load are transport workers and that includes road drivers [2-5] as well as seafarers. Recent studies from Poland, France, Norway, Germany and Denmark have reported that high blood pressure, high triglycerides, diabetes and obesity as well as risk behaviors such as smoking and physical inactivity are not only highly prevalent in seafarers [6-13] but also are much more common than among respective general populations [9,10,14,15].

Besides the fact that seafaring is still dominated by a “male lifestyle-culture”, which might explain the comparatively high smoking rates and unhealthy, high-fat eating habits; a main reason for such differences might also be found in the specific environmental conditions encountered by seafarers. On-board periods are often long and leisure time choice of activities is usually limited. Much of the leisure time is thus spent on meals, snacking, resting and corresponding with family/friends, whereas only a minority of employees engage in physical fitness activities [10]. The confined space on board makes the most common choice of exercise for people on shore, that is running/walking, impossible, and the constant change between longer periods on board and longer periods at home might make it hard to establish important routines for exercise. Combined with the fact that many jobs on modern vessels have become largely sedentary or require only moderate levels of energy expenditure, the extent of physical inactivity when at work among this occupational group is alarmingly high [10]. Furthermore, nutrition quality is often limited by the fact that for smaller ships, companies tend not to employ professional cooks but to shift cooking duties among the crew. This often results in a stronger reliance on traditional high-fat, high-sugar foods with lesser emphasis on healthy eating.

Since the work place creates many of the conditions, which promote or reinforce unhealthy behavior, it also provides an important arena for health interventions with the advantage that larger populations can be reached in a place where they spend a lot of time and where individuals can be targeted within their social networks of coworkers [16]. To get the full benefit of work place health promotion (WHP) efforts, it is essential to conceive of these as combined activities of employers, employees and societies. This is “*…achieved by a combination of improved work organization and work environment, improved support for workers ‘personal development and promotion of employees’ active participation”*[17]*.* Many studies on the effects of land-based WHP and WHP intervention programs have shown that health behavior as well as health can be improved [16], however only one such study exists within the maritime setting. This one-year follow-up study from Finland aimed to activate sailors to take care of their own health and well-being by way of health education courses as well as environmental changes of e.g. exercise equipment on board. Results revealed an increase in the frequency of physical exercise at sea as on shore, meals were perceived as better and healthier at the intervention’s follow-up, but no changes were found in physiological parameters, such as blood lipids and blood pressure [13].

Thus, there is a definitive need for further studies to investigate the effects of different types of health promotion interventions at sea as well as for studies to provide knowledge on how to best implement such interventions in the maritime industry and how to maintain them.

### Aim

The overall aim of this study was to contribute new knowledge to the evidence base on the effects of a maritime health promotion intervention as well as the challenges encountered in implementing such an intervention.

### Objectives

Based on a one-year follow-up study of seafarers in two Danish shipping companies the objectives were to:

1. Identify changes in lifestyle related risk behaviors, such as smoking, physical (in) activity and unhealthy eating from a structural and- and/or health education intervention,

2. Identify changes in the prevalence of high physical fitness, high waist circumference as well as metabolic syndrome related to a structural- and health education intervention,

3. Identify challenges in the implementation process of a health promotion program applied in the maritime work place.

## Method

### Study design and procedure

**Figure 1:**
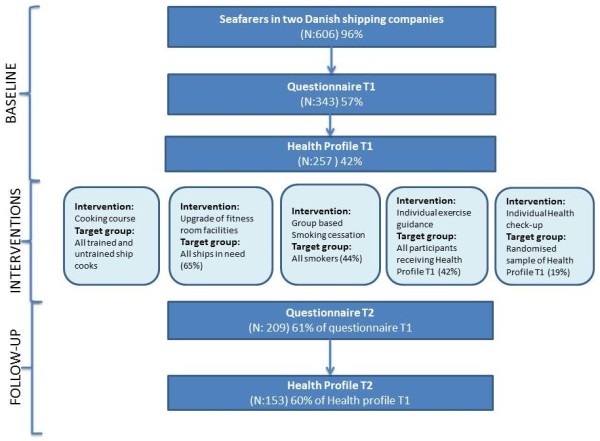
Flowchart of the participant flow from baseline to follow-up.

The study was based on a single-group pre-post design measuring lifestyle related risk behaviors and diverse health risk factors among seafarers in two Danish shipping companies before and after implementing several health promotion interventions. Baseline and follow-up data were collected with the help of 1) a self-administered standardized questionnaire, which was sent to all employees of the two companies, and 2) an individual health profile that measured parameters of physical health and physical fitness. The questionnaire was posted end of 2007 and again approximately one year after, in the beginning of 2009, to the home address of all seafaring employees (N = 630). For the follow-up an electronic version of the questionnaire was also made available. The health profile was carried out between October 2007 and December 2008, and follow-up data were collected between January and December 2009. In addition, qualitative interviews were conducted face-to-face or by phone to gain knowledge about the reasons of those who were eligible but did not participate in the different health education intervention modules. The three phases in the study design and the participant flow through these phases are illustrated in the flowchart below (Figure 1).

### Study sample

The participating shipping companies consisted of a cargo service company, which operated mainly in the North Atlantic between Aalborg in Denmark and Greenland’s Disco Bay and had approximately 190 seafaring employees. The majority of these seafarers were nationals of Denmark and Greenland. The off-shore period was four to eight weeks, followed by four to eight weeks at home. The average crew size was 12 to 15 people. The work consisted mainly of cargo management during the port visits and maintenance of the ship. The second company was an offshore rescue and support vessel enterprise, which mainly operated in the North Sea, where they circulated offshore installations, keeping watch for accidents such as oil spill or “man overboard” incidents. The company had approximately 440 employees, the vast majority were nationals of Denmark and the Faroe islands. The off-shore period was two to four weeks, followed by two to four weeks off. Crew size varied from 6 to 12 people. Aside from maintenance of the ship, the crew’s main task was to practice regular rescue and security drills. The mean age of the sample at T1 was 42 years (SD 10.5) and 63% of all participants were officers (see Table 1). Since 95% of respondents were male (which reflected the gender distribution among the employees), it was decided to restrict all further analysis to this male subsample.

**Table 1:** Baseline, follow-up and drop-out characteristics of male seafarers in two different shipping companies obtained through questionnaires and health examinations

	**Total**			**Company 1**			**Company 2**		
**Questionnaire data**	**Baseline questionnaire T1**	**Follow-up questionnaire T2**	**Drop-out**	**Baseline questionnaire T1**	**Follow-up questionnaire T2**	**Drop-out**	**Baseline questionnaire T1**	**Follow-up questionnaire T2**	**Drop-out**
	**(N = 343)**	**(N = 209)**	**(N = 134)**	**(N = 89)**	**(N = 52)**	**(N = 37)**	**(N = 254)**	**(N = 157)**	**(N = 97)**
	**N (%)**	**N (%)**	**N (%)**	**N (%)**	**N (%)**	**N (%)**	**N (%)**	**N (%)**	**N (%)**
Age (M/SD)	42 (10.5)	44 (10.3)	41 (10.4)	41 (10.7)	42 (10.5)	40 (11.1)	43 (10.3)	45 (10.4)	41 (10.2)
Men	343 (95%)	209 (96%)	134 (94%)	89 (85%)	52 (88%)	37 (80%)	254 (100%)	157 (99%)	97 (100%)
Officer rank	214 (63%)	148 (71%)	77 (59%)	65 (75%)	42 (81%)	24 (69%)	149 (59%)	106 (68%)	53 (56%)
Smokers	144 (44%)	75 (37%)	64 (49%)	36 (42%)	15 (29%)	16 (43%)	108 (45%)	60 (39%)	48 (52%)
Frequency of exercise level ≥ 3 times weekly at home	82 (24%)	57 (28%)	30 (23%)	16 (18%)	12 (23%)	6 (17%)	66 (27%)	45 (30%)	24 (25%)
Frequency of exercise level ≥ 3 times weekly at sea	108 (32%)	73 (35%)	42 (32%)	16 (19%)	13 (25%)	6 (17%)	92 (37%)	60 (39%)	36 (37%)
Frequency of exercise level < 1 time active weekly or never at home	165 (49%)	93 (46%)	64 (50%)	47 (54%)	28 (54%)	18 (50%)	118 (48%)	65 (43%)	46 (50%)
Frequency of exercise level < 1 time active weekly or never at sea	164 (49%)	83 (40%)	72 (55%)	50 (58%)	27 (52%)	22 (63%)	114 (45%)	56 (36%)	50 (52%)
Frequency of overeating ≥ 3 days weekly at home	133 (40%)	79 (38%)	49 (39%)	37 (43%)	19 (37%)	15 (42%)	96 (40%)	60 (39%)	34 (37%)
Frequency of overeating ≥ 3 days weekly at sea	152 (47%)	86 (42%)	64 (51%)	41 (47%)	25 (49%)	18 (50%)	111 (47%)	61 (40%)	46 (51%)
Frequency of eating high-sugar products ≥ 3 days weekly at home	132 (40%)	61 (30%)	49 (38%)	37 (43%)	14 (27%)	15 (42%)	95 (39%)	47 (31%)	34 (37%)
Frequency of eating high-sugar products ≥ 3 days weekly at sea	170 (52%)	86 (43%)	65 (50%)	46 (53%)	23 (44%)	19 (53%)	124 (52%)	63 (42%)	45 (49%)
	**Total**			**Company 1**			**Company 2**		
**Health profile data**	**Baseline health profile T1**	**Follow-up health profile T2**	**Drop-out**	**Baseline health profile T1**	**Follow-up health profile T2**	**Drop-out**	**Baseline health profile T1**	**Follow-up health profile T2**	**Drop-out**
	**(N = 257)**	**(N = 153)**	**(N = 104)**	**(N = 75)**	**(N = 49)**	**(N = 26)**	**(N = 182)**	**(N = 104)**	**(N = 78)**
High physical fitness (age and gender standardized VO2submax test)	69 (30%)	71 (50%)	24 (26%)	19 (33%)	23 (52%)	6 (33%)	50 (29%)	48 (49%)	11 (24%)
Obesity (≥ BMI 30)	64 (25%)	42 (28%)	25 (24%)	20 (27%)	14 (29%)	10 (39%)	44 (24%)	28 (27%)	15 (20%)
High waist circumference (wc), male ≥94 cm	163 (66%)	96 (65%)	58 (59%)	52 (75%)	33 (72%)	16 (70%)	111 (62%)	63 (63%)	42 (55%)
Metabolic syndrome (wc ≥94 cm and 2 further risk factors)	123 (50%)	56 (37%)	44 (42%)	41 (55%)	24 (49%)	12 (46%)	81 (45%)	32 (31%)	32 (41%)

A total of 606 male seafarers were invited to take part in the study by filling in the first questionnaire at T1 (QT1), which 57% of the seafarers did. Of these, 61% also completed questionnaire T2 (QT2). The drop-out rate between QT1 and QT2 was 39%, and the overall response rate for participating in both questionnaire rounds relative to the entire employee population was 35%. Health profile 1 (HPT1) was received by 42% of the employees. Of these 60% also received the follow-up health profile T2 (HPT2), which amounts to a drop-out rate of 40%. The response rate for completing health profiles 1 and 2 was 25%. Comparisons of different characteristics between baseline, follow-up participants and drop-outs showed no major differences at baseline (see Table 1). Qualitative interviews were conducted with 21 participants who dropped out between HPT1 and HPT2.

### Interventions

Five different interventions were implemented in 2008/2009. Two of these were structural or socio-ecological interventions aimed at providing a healthier environment for all seafarers in the two companies. One of the interventions was a two-day course on healthy cooking for all chefs and staff with cooking responsibilities which was run at five alternate days between May 2008 and January 2009. The first day offered an introduction to healthy diet according to official Danish recommendations and how to improve nutrition in everyday meals. A specific target was reduction of sugar and fat, e.g. serving fruit and healthy snacks instead of cake at the daily coffee breaks. The second day was devoted to motivation and communication skills needed for gaining acceptance for a more nutritious diet on board the ships. The course took place at the facilities of a leading supplier of catering equipment, which offered the opportunity to cook with the newest and most advanced equipment during the practical cooking tasks. It was led by a nutrition expert with extensive experience in the maritime setting. All participants received a set of course documents, containing the recipes and handouts of the lectures. The other effort at changing environmental structures related to availability of/accessibility to high-quality exercise equipment and involved the modernization and upgrade of the fitness rooms’ equipment on board the ships.

In addition, three health education interventions were offered. One was a group-based smoking cessation course targeted at all smokers among the employees. Everybody who had expressed a wish to participate in a cessation course in questionnaire T1 or the health profile T1 was offered such a course including counseling on quitting as well as lung function tests and guidance on and reimbursement of nicotine replacement products. Group counseling was led by a trained nurse specialized in smoking cessation and consisted of two two-hour meetings scheduled within a three-week period onshore in the respective home ports of the two companies. The first meeting aimed at strengthening motivation as well as help with concrete preparation for cessation, the second was supposed to support coping with withdrawal symptoms and craving. The meetings were supplemented by three individual follow-up telephone contacts. All seafarers who had shown interest in such a course were contacted via normal as well as electronic mail and offered two alternative dates in May 2008. Those who did not reply received another e-mail invitation, and if there again was no reply were called up by phone to be personally invited once more.

The second health educational initiative related to exercise training. All participants receiving the individual health profile at T1 were offered motivational counseling in combination with individual guidance on physical training with a physiotherapist specialized in exercise training. The offer consisted of a tailored program based on the individual seafarers’ needs taking into account the results of their individual health profile and the physical problems indicated therein, such as metabolic syndrome or muscular-skeletal disorders. The individual programs offered printouts with pictures for each exercise element to enable home training. Each participant would afterwards be able to go ahead with his/her program to e.g. improve fitness, lose weight, or reduce joint and muscle pains and was additionally offered the option of a three-month follow-up session. A competition was held in the project period organized by the Committee on Seafarers Welfare for seafarers in the Danish merchant fleet, doing most kilometers on fitness bikes at sea on an individual basis or collectively by ship.

The third and last health education intervention was an extra health check-up with health feedback. From the group of seafarers, who had received the baseline health profile, 50 were randomly selected from the participant list to be offered three extra health-check-ups every three months, consisting of the same anthropometric and physiological measurements, which were offered in the first health profile. The aim of this component was to find out if a closer monitoring and continuous feedback had any impact on the seafarers lifestyles beyond the initial health information they received by the first health check-up for all.

### Measurement

#### Standardized questionaire

The questionnaire covered seafarers’ perceived health, well-being, and health-related behaviors. It consisted of one open and 68 closed questions with standard rating scales. Smoking status was assessed by asking: “How many cigarettes do you smoke per day – on average?” with the following reply options: “none”, “1-5 cigarettes”, “6-10 cigarettes”, “11-15 cigarettes” or “more than 15 cigarettes”. From these a dichotomous variable was created defining non-smokers as those who smoked no cigarettes, smokers as those smoking one or more cigarettes per day. Assessment of frequency of physical activity at sea and at home was based on one question each for the sea and the home settings: “How often do you exercise, so it increases your fitness and/or strengthens muscles?” Response options were: “3 times a week or more”, “1-2 times a week” “Less than once a week” and “Never”. In accordance with official recommendations, insufficient or low physical activity was defined as 2 times a week or less, while ≥3 times a week was considered sufficient or high physical activity. In relation to eating habits at sea and at home, two questions were asked, one for frequency of overeating (“Do you eat more than you need?”) and one for intake of sugared products: (“Do you eat cake, sweets/drink sugared sodas?“). Response options were: “5-7 days a week”, “3-4 days a week”, “1-2 days a week” and “less than 1–2 days a week”. Low intake of sugar was defined as 2 times a week or less, while ≥3 times a week was defined as high sugar intake.

### Individual health profiles: anthropometric and cardiovascular fitness measurement

Anthropometric and cardiovascular fitness measurements were recorded by a registered nurse and/or by a physiotherapist during the course of individual sessions on board and on land.

Fitness was assessed from the sub-maximal exercise test using a cycle ergometer and pulse meter to estimate maximal oxygen uptake (VO2max) based on two consecutive workload intervals, divided by body weight in kg. Fitness scores were divided into three groups: low, medium and high stratified for age and gender [18]. Cut off for low or high fitness score was low-medium versus high.

A BMI-of 30 and above was used as an index of general obesity [19]. Waist circumference was measured between the lowest rib and the top of the person’s hipbone. A WHO-recommended circumference of 94 cm for males was chosen as cut-off to distinguish normal from enlarged sizes [20]. Blood pressure was measured in millimeters of mercury (mm Hg) with an inflatable cuff on the upper arm (“Omron M7” over-arm devices). All measurements indicating high blood pressure were repeated at the end of the session to reduce effects of nervousness (white coat hypertension). Cholesterol and plasma glucose was measured by way of a prick test in the finger (“Cholestec LDX” equipment, which is a lipid analyzer providing results after just few minutes). Presence of metabolic syndrome was defined in accordance with the guidelines of the International Diabetes Federation (IDF): Central obesity of ≥ 94 cm for males plus presence of at least two of four other risk factors: Raised triglycerides (≥1.7 mmol/L for males), reduced HDL cholesterol (<1.03 mmol/L for males), raised blood pressure (systolic BP ≥ 130 or diastolic BP ≥85 mm Hg) and raised fastening plasma glucose (≥5.6 mmol/L) [21].

### Data analyses

To describe health behaviors and health status indicators at T1 and T2, mean, standard deviations and percentages were used. To determine change in behaviors and health status indicators between baseline and follow-up, cross tabulations with percentages and McNemar tests were used, matching pairs of subjects between the two periods of time. Results are presented as McNemar p-values. A level of p < .05 was regarded as statistically significant. Associations between participation in the individual health education intervention modules (smoking cessation course, exercise guidance and/or extra health profile) and the various criterion variables were tested with hierarchical logistic regression analyses entering the appropriate intervention(s) as covariates into the equation after in a first step adjusting for the baseline of the respective criterion variable, age and rank (officers versus crew). Results are presented as Odds ratios (OR) with 95% confidence intervals (CI). P < .05 was regarded as statistically significant. All statistical analyses were performed using IBM SPSS version 20.

### Ethical considerations

According to the Danish National Committee on Health Research Ethics no ethical approval was required. The study design including data handling, anonymization and storage procedures were reported to the Danish Data Protection Agency. All respondents were informed about the aim of the study and were included in the study after having provided verbal and written informed consent.

## Results

### Changes in health behaviors and health indicators from T1 to T2

Table 1 shows the percentages for socio-demographic characteristics as well as prevalence rates for the different health/health behavior indicators at T1 and T2 including the drop-outs. Table 2 presents prevalence rates only for the subgroup of those who provided valid data at T1 and T2.

**Table 2:** Prevalence of life-style behaviors and risk factors at T1 and T2

	**Health hehaviors and health indicators**	**T1**	**T2**	**p**
		**N (%)**	**N (%)**	
	Smokers	79 (40%)	74 (38%)	0.300
At home	Physical exercise ≥ 3 times weekly	50 (25%)	53 (27%)	0.749
At sea	Physical exercise ≥ 3 times weekly	65 (32%)	72 (35%)	0.435
At home	Physical exercise < 1 weekly or never	100 (50%)	93 (46%)	0.390
At sea	Physical exercise < 1 weekly or never	92 (45%)	82 (40%)	0.250
At home	Overeating ≥ 3 times weekly	83 (42%)	78 (39%)	0.576
At sea	Overeating ≥ 3 times weekly	85 (45%)	83 (43%)	0.883
At home	Intake of high-sugar products ≥ 3 times weekly	81 (41%)	60 (30%)	**0.004**
At sea	Intake of high-sugar products ≥ 3 times weekly	102 (53%)	84 (44%)	**0.022**
	High fitness score	45 (34%)	67 (51%)	**0.000**
	High waist circumference ≥ 94 cm	102 (71%)	93 (65%)	0.064
	Metabolic syndrome	79 (57%)	66 (48%)	**0.029**

### Smoking

The overall percentage of smokers in the study sample decreased from 40% to 35% between T1 and T2, which was non-significant (see Table 2). When participants versus non-participants in the smoking cessation course were compared, a significant effect for participation was found (see Table 3). Thirty-three percent of the participants in the smoking cessation intervention had quit smoking at T2 compared to only eight percent of the seafarers who had not participated in the intervention.

**Table 3:** Intervention participation and smoking

	**Smoking at T2**	
	***N = 73***	
	OR	(CI)
Age^1^	0.96	(0.90-1.03)
Rank^2^	0.33	(0.06-1.74)
Intervention		
Smoking cessation course^3^	0.13	(0.02-0.81)

^1^Cont. variable, ascending; ^2^Officers = 1, Non-officers = 2; ^3^no = 0, yes = 1.

### Exercise activity and fitness score

As for the target of reaching officially recommended levels of exercise activity (3 times and more a week) only slight increases of 2% (at home) and 3% (at sea) from T1 to T2 were found for the overall group. To additionally check whether changes below that level of high activity occurred, analyses were also performed for moving from being largely inactive (0–1 times a week) to being active more than once a week. In this case 4% less were inactive at home and 5% less at sea, however, this was again not significant (see Table 2). Neither was there any significant association between participating in the exercise counseling or receiving an extra health check and the level of exercise activity or inactivity at follow-up (Table 4). However, the share of seafarers with a high fitness score increased significantly from 34% at T1 to 50% at T2 (see Table 2), although no significant relation to participating in the exercise guidance or the extra health profile was found (see Table 4).

**Table 4:** Intervention participation and high exercise level/high physical fitness score at T2

	**High exercise level at home (T2)**		**High exercise level at sea (T2)**		**High physical fitness score(T2)**	
	**(Thrice a week or more)**		**(Thrice a week or more)**			
	***N = 146***		***N = 149***		***N = 126***	
	**OR**	**(CI)**	**OR**	**(CI)**	**OR**	**(CI)**
High physical exercise level T1^1^	10.50	(4.11-26.8)	3.56	(1.68-7.58)		
High physical fitness score T1^2^					7.34	(2.97-18.17)
Age^3^	1.02	(0.98-1.06)	1.05	(1.01-1.09)	1.01	(0.97-1.04)
Rank^4^	0.44	(0.16-1.18)	0.47	(0.21-1.07)	0.50	(0.21-1.16)
Interventions						
Physical exercise guidance^5^	1.10	(0.43-2.83)	1.06	(0.47-2.38)	1.26	(0.54-2.93)
Extra health check-up^6^	1.39	(0.44-4.36)	1.10	(0.40-3.06)	0.60	(0.22-1.65)

^1^≤2 times a week = 0, ≥3 time a week = 1; ^2^low = 0, high = 1; ^3^Cont. variable, ascending; ^4^Officers = 1, Non-officers = 2; ^5^no = 0; yes = 1; ^6^no = 0, yes = 1.

### Dietary behavior

There was no significant reduction in the self-reported tendency to overeat at sea or at home between T1 and T2 (Table 2). However, for both, the sea and the home setting the percentage of study participants reporting frequent intake of high-sugar products, such as sweets, cake or sodas had decreased significantly. Logistic regression analysis indicated that this change in eating behavior was not influenced by participation in the extra health profile (Table 5).

**Table 5:** Intervention participation and dietary behavior at T2

	**Overeating**^**1 **^**at home (T2)**		**Overeating**^**1 **^**at sea (T2)**		**Eating high-sugar products at home (T2)**		**Eating high-sugar products at sea (T2)**	
	**(≥Three days a week)**		**(≥Three days a week)**		**(≥Three days a week)**		**(≥Three days a week)**	
	***N = 155***		***N = 147***		***N = 153***		**N = 147**	
	**OR**	**(CI)**	**OR**	**(CI)**	**OR**	**(CI)**	**OR**	**(CI)**
Overeating T1^1^	6.63	(3.23-13.61)	8.10	(3.85-17.02)				
Eating high-sugar products T1^1^					8.77	(3.81-20.20)	6.69	(3.11-14.39)
Age^2^	1.01	(0.97-1.04)	1.01	(0.97-1.05)	0.97	(0.94-1.01)	0.97	(0.93-1.00)
Rank^3^	0.75	(0.35-1.62)	0.77	(0.35-1.68)	0.38	(0.24-1.36)	1.13	(0.52-2.44)
Interventions								
Extra health check-up^4^	0.63	(0.22-1.80)	1.06	(0.36-3.10)	1.50	(0.52-4.30)	1.11	(0.40-3.09)

^1^≤2 days a week = 0, ≥3 days a week = 1; ^2^Cont. variable, ascending; ^3^Officers = 1, Non-officers = 2; ^4^No = 0, yes = 1.

### Waist circumference and metabolic syndrome

The percentage of those with high waist circumference had decreased by 5% from 71% at T1 to 66% at T2. This was, however, only a non-significant trend. For metabolic syndrome on the other hand there was a significant decrease from 57% to 48% of affected seafarers between T1 and T2 (see Table 2). In none of these cases was there any significant association between participating in the exercise guidance or the extra health profile interventions and the respective outcomes (Table 6).

**Table 6:** Intervention participation and metabolic syndrome at T2

	**Metabolic syndrome T2**	
	***N = 131***	
	**OR**	**(CI)**
Metabolic syndrome T1^1^	14.79	(5.88-37.19)
Age^2^	1.00	(0.96-1.04)
Rank^3^	1.12	(0.47-2.70)
Interventions		
Physical exercise guidance^4^	0.85	(0.35-2.04)
Extra health check-up^5^	0.52	(0.18-1.53)

^1^No = 0, yes = 1, ^2^Cont. variable, ascending; ^3^Officers = 1, Non-officers = 2; ^4^No = 0; yes = 1; ^5^No = 1, yes = 2.

### Implementation of the intervention components and participant reach

As for the *smoking cessation course*, about half (49%; N = 70) of all employees who smoked indicated that they were interested in a cessation course at T1. Of this group only 13, that is 18% of the motivated subgroup, actually joined one of the two offered courses. Furthermore, only one of these courses actually ran both of the initially planned group meetings, while the other course had to cancel the second meeting due to an inability to find a commonly acceptable date. According to the qualitative interviews with non-participants of the course the low attendance rate was due mainly to logistical issues. Among these were foremost conflicting sailing schedules, which meant it was impossible to find meeting dates fitting the schedules of all crew members from different ships. Another often mentioned issue was that seafarers’ home bases were geographically dispersed and that family obligations during the home period prevented long transportation times back to course localities in port.

Thirty percent (N = 76) of those eligible (all who took part in HPT1), accepted the offer to receive *exercise guidance* and of these 37% (N = 28) also received the 3-months follow-up guidance. Responses from interviews with non-participants indicated communication problems since some did not recall being offered the intervention at all. Other reasons given were mainly either that participants felt healthy/and or that they were already physically active and had sufficient knowledge of how to use the fitness facilities.

The target group for the *extra health check-ups* consisted of 50 seafarers who had been randomly selected from the subgroup of those who had received the first health profile. Only 27 of these (54%) took up the offer. Reasons given for non-participation were mainly related to logistics such as conflicting sailing schedules and – during home leave – distances too far from the locations where the physical exams were scheduled. If the location of the office was thus not in a convenient distance of the seafarers whereabouts at the given date of the check-up, they were inclined to reject participation.

The *cooking course*, which was announced as mandatory by the companies, was attended by 49 ship cooks, which equals 75% of all cooks in the two companies. Reasons for non-participation were again mainly conflicting sailing schedules.

An *upgrade of fitness room facilities* onboard the ships was requested by 64% of the participating ships (N = 20) and in the individual interviews with seafarers from the different ships 14 (70%) reported that improvements had been made.

## Discussion

This study is among the first investigations of health promotion interventions in the maritime work place. Work place health promotion in general, has often proved to be challenging [22], but the maritime setting seems even more demanding than most. According to two systematic reviews, typical, worksite intervention studies have reported participation rates as low as 8-10% and as high as 64-97% with a median of 33-61% [23-25]. Rates for the present study were above the lower limit and – at least in comparison to one of the systematic reviews – above the reported median. Initial interest in participation in the different intervention offers varied between around 30-50%. Only the semi-mandatory cooking courses reached a rate of 75%. This might partly reflect a general lack of motivation or prioritization of health issues among seafarers or a reluctance to deal with these issues in the work place (see below), which suggests that considerably more efforts at motivating this target group for health promotion and marketing such interventions might be called for. However, it also became clear that actual reach was still considerably below the initial rates, and a main factor for this seems to lie in the nature of the work. Seafarers and their work places literally are “moving targets” where not only the work places (the ships) travel, but seafarers also frequently shift between ships and all move from ships to their homes which are widely dispersed which creates fundamental different changes from “normal”, land-based WHP. Providing interventions for such a target group is a distinct logistic challenge which might require resources beyond the level of what can be expected to be needed for a “normal” stationary work place.

### Changes in health behaviors and health indicators

As for percentage of *smokers* among employees, only a slight and non-significant decrease of 2% occurred between both measurement points. Considering that between 2008 and 2009 a 7% reduction of daily smokers was registered among men from the general Danish population aged 20–69 years, seafarers not only had higher smoking rates than the general male population [26,27], but also seemed to be lagging behind the downward secular trend It should be noted, however, that such a comparison is necessarily tentative since possible differences in educational and occupational backgrounds between the two samples cannot be accounted for. A significant positive effect was found for the smoking cessation intervention, which, however, should be interpreted with caution, as only few seafarers had signed up for the course. While for many non-participants logistic issues seemed to have played a major role for not feeling able to attend, it must also be assumed that the few who actually did attend differed substantially in motivation and determination from those who did not. However, it also needs to be noted that the finding is in line with results from a Cochrane review of onshore workplace interventions for smoking cessation [28]. Cahill et al. [28] found strong evidence that individual workplace cessation interventions as well as group counseling and pharmacological treatment to overcome nicotine addiction significantly increased the likelihood of quitting smoking. Despite its methodological limitations the present study indicates that a smoking cessation intervention in the maritime workplace setting has the potential to make a significant contribution to seafarers’ health. To achieve more broad-based success, a more specified and tailored approach is required which takes into account the specific restrictions inherent in a “moving work place”. Instead of trying to schedule joint dates for crew members from different ships, which seems non-feasible, it might be tried to target smokers within their ship crews in order to ensure some continuity of group counseling and also enable daily group support by offering sessions compatible with arrival or departure times in/from port, by sending out counselors to the ships while in port or during crew change at sea and/or by offering internet-based support.

Even though slight improvements on *exercise level* were noted, these changes were not significant, whereas there was a significant increase in *physical fitness scores* showing that 1/3 of the participants had improved their fitness score at T2 towards the recommended level. One explanation for this seeming discrepancy might be a difference in samples, since the fitness scores could only be computed for the smaller – and probably more motivated – subsample, which had not only participated in the questionnaire survey but also in the health profile at baseline and follow-up. However, an additional analysis of exercise change for this subgroup yielded the same non-significant result as for the larger sample. Another reason may be that measurement of exercise behavior in terms of frequency without including a measure of duration/intensity might have prevented accurate classification and underrated possible changes.

There was no differential change from T1 to T2 based on participation in the exercise guidance or the extra health check-up. As described, the one-dimensional measurement of exercise behavior might have prevented detecting change, in particular as the exercise guidance emphasized adequate fitness training and correct use of fitness equipment which might be expected to impact duration or intensity of exercise rather than frequency alone. As for the extra health risk check-up it could be discussed whether the first health profile that was used for baseline assessment of fitness and health parameters and was provided to all might not already have motivated participants into contemplating change, so that the additional monitoring of health status three months later was not able to make a substantial additional contribution. A further aspect to be taken into account is the partial implementation failure. Due to the substantial drop-out, both the exercise guidance and the health-check were implemented as single events and not as a monitoring system providing feedback in regular intervals. Yet another factor could be program failure. A recent review by Vuillemin et al. [29] on general worksite physical activity interventions reported moderate evidence for effects of longer-term exercise training programs on physical fitness outcomes and exercise behavior but inconclusive or lacking evidence for counseling interventions. Exercise guidance and individual feedback about health and fitness status are both counseling-type components and it might be discussed whether more intense and longer-term guided exercise programs which create socially more binding structures are likewise required in the maritime setting. This might be more difficult to achieve for seafaring than for onshore workplaces, but web-based communication devices might be considered for overcoming logistic problems

Beyond the lack of evidence for effects of the health education modules, it needs to be noted that the change which occurred in fitness might be attributable to the structural changes made by upgrading fitness rooms on board in combination with the treadmill/rowing machine competitions between boats. In a similar vein, a Finnish study on seafarers with high risk factor load installed new fitness rooms on board or improved fitness room equipment, provided exercise guidance and subsidized fitness club visits on shore and found a 25% decrease in inactivity from baseline to the one-year-follow-up [13].

As for *dietary behavior*, there was no significant change in reported overeating, while intake of high-sugar products, such as sweets, cake and sodas decreased significantly between T1 and T2. This change was not associated with participating in an additional individual health monitoring, but it might be assumed that the “healthy cooking courses” offered to ship cooks might at least have contributed to this development. As no control group was assigned, no definite effect attribution is possible. However, additional findings from interviews with the participating cooks, which have been reported elsewhere [10,23] and in Hjarnoe, L. and Leppin, A.: What does it take to get a healthy diet at sea? A maritime study of the challenges of transferring knowledge from a health promotion intervention to the workplace at sea, submittet] suggest that on many ships supply changes were made in terms of reducing fat and sugar content in meals, offering fruit instead of cake and/or abolishing sugared soda drinks.

The Finnish study on health promotion for seafarers similarly introduced training for ship cooks in preparing lighter meals combined with group interventions such as “weight-watcher” groups and individual support from occupational nurses. Seafarers perceived the meals at the one-year-follow up as being healthier than at baseline. Similarly, two recent reviews on general worksite health promotion interventions for employees’ diets found evidence for small to moderate effects of educational and/or structural interventions, particularly for fruit, vegetable and fat intake [22,29].

Beyond self-report changes in behavior, the most notable was a significant decrease in the percentage of employees with *metabolic syndrome*. Again, there was no significant association of this change with participating in the extra health risk feedback or in the exercise guidance. Like for the decrease in self-reported intake of high-sugar products, the positive change in the meals served on board due to the cooking intervention for ship cooks might have contributed to this development. In fact, additional sub-analyses (not reported here) showed that the risk factor for metabolic syndrome, which had changed most, was glucose level. This is in contrast to the Finnish study on work site health promotion among seafarers [13] which also reported improvements in self-reported eating behavior, but did not find changes in related physiological parameters. Both studies were based on pretest-posttest designs though, which clearly restricts internal validity. Also, it is important to note that metabolic syndrome still was highly prevalent in the sample. A US-American cross-sectional study of health characteristics among merchant marine captains and pilots showed similar rates of 39% with metabolic syndrome [30]. In comparison, a Danish study of the general population found only 20% of 20–97 year old males with metabolic syndrome [31], and a Canadian study from 2011 revealed 18% prevalence among its male participants [32]. In particular, when assuming a healthy-worker effect due to the requirements of frequent health examinations, the rates among seafarers are alarming and indicate an urgent need for intensified intervention efforts.

### Study limitations

A major limitation of the study is the possibility of selection bias. 43% of employees did not participate in the first questionnaire round and even more did not take part in the first health profile (58%). Interviews with non-participants of health profile 1 revealed different motives, some of which suggest more random effects, such as misunderstandings about locations or time frames for signing up as well as conflicting sailing schedules. Other explanations, however, indicate more systematic influences, such as seeing lifestyles as a matter of privacy or being afraid that data would be registered and followed by employers, but also feeling no need for personal participation due to an activity status which was already perceived as high. Furthermore, there were sizeable drop-out rates towards T2 of 39% for the questionnaire survey and 40% for the health profile. There did not appear to be substantial differences in the main variables of interest between these two groups at baseline, but it can be expected that a substantial part of the drop-outs were those who did not improve over time so that some of the more favorable developments found might be overestimations. This difficulty to attribute positive changes over time to one or several of the various interventions is furthermore reinforced by reliance on a before-after design without a randomized or in fact any control group. This lack of control group was due to the specific work organization process where crews shifted between ships on a regular basis which made a fixed assignment of crews/ships to an intervention or control condition not feasible. For the health education interventions it was possible to compare participants with non-participants. Such comparisons, however, are naturally problematic due to non-equivalence of the groups, not at least due to differences in motivation to change.

There certainly is a need for more methodologically rigorous studies, but it also needs to be noted that there is a genuine conflict between demands for scientific rigor and stakeholder needs for and interests in workplace interventions [22]. Moreover, the organization of the maritime setting in particular presents practical challenges, which makes control group designs technically hard to achieve due to constantly moving work places and many crew members regularly shifting ships. Lastly, limitations in behavior measurement need to be acknowledged. Like in all studies using self-report measures, reporting bias might have occurred due to social desirability tendencies. Another pertinent problem might be a lack of differentiation in measurement as already discussed for exercise assessment. Similarly, food diaries or more elaborate food questionnaires might have provided more reliable and valid results than single items asking for frequency of consuming different types of food.

## Conclusions

The study contributes with new knowledge about health promotion in a work place which is special, but important for many trade-dependent economies: seafaring. One of the major findings was that implementing health promotion interventions in the maritime workplace setting is a challenging task due to the “moving nature” of the maritime work place, which makes implementation, particularly a high participant reach difficult to achieve. Future studies in the field need to focus on this aspect and try to make efforts at ensuring sufficient and sustainable reach for intervention implementation. Because of methodological limitations in the design of the study the evidence of changes found in behavior and health parameters (smoking, fitness and metabolic syndrome) in the present study should be interpreted with caution. Also, effects could not be clearly attributed to any specific type of intervention due to the multicomponent nature of the program. The specific contributions of different intervention types certainly need further investigation. Particularly the involvement of the employers, i.e. the companies and their engagement in health promotion initiatives on a structural level, such as, for instance, training ship cooks in providing healthier meals for all or upgrading fitness rooms on board might have influenced health and lifestyle changes of the seafarers. However, even after such changes were initiated, prevalence of risk factors was still high suggesting a need for increased efforts aimed at easily accessible and specifically tailored health promotion initiatives, preferably in line with ‘safety management systems’, taking into account the special conditions of the maritime workplace setting.

## Competing interests

The authors declare that they have no competing interests.

## Authors’ contributions

The authors declare that both have satisfied the authorship requirement to this manuscript in accordance with the Vancouver regulations. LH was in charge of data collection and coding. LH and AL participated in the analysis and interpretation of data. LH wrote the first draft. Both authors performed critical revisions of drafts and read and approved the final manuscript.

## Pre-publication history

The pre-publication history for this paper can be accessed here:

http://www.biomedcentral.com/1471-2458/13/1165/prepub
